# Diagnostic Significance of Serum HMGB1 in Colorectal Carcinomas

**DOI:** 10.1371/journal.pone.0034318

**Published:** 2012-04-04

**Authors:** Hanna Lee, Meiying Song, Nara Shin, Chang Hoon Shin, Byung Soh Min, Hyon-Suk Kim, Jong Shin Yoo, Hoguen Kim

**Affiliations:** 1 Department of Pathology, Yonsei University College of Medicine, Seoul, Korea; 2 Brain Korea 21 Project for Medical Sciences, Yonsei University College of Medicine, Seoul, Korea; 3 Department of Surgery, Yonsei University College of Medicine, Seoul, Korea; 4 Department of Laboratory Medicine, Yonsei University College of Medicine, Seoul, Korea; 5 Division of mass spectrometric analysis, Korea Basic Science Institute, Cheongwon, Korea; University of Munich, Germany

## Abstract

High mobility group box 1 protein (HMGB1), a nuclear protein, can be translocated to the cytoplasm and secreted in colon cancer cells. However, the diagnostic significance of HMGB1 has not been evaluated in colorectal carcinomas. For this purpose, we have screened the expression and secretion of HMGB1 in 10 colon cancer cell lines and 1 control cell line and found that HMGB1 was detected in the culture medium. To evaluate the diagnostic value of HMGB1, we performed an enzyme-linked immunosorbent assay to measure HMGB1 levels and compared them to carcinoembryonic antigen (CEA) levels in the serum samples of 219 colorectal carcinoma patients and 75 healthy control subjects. We found that the serum HMGB1 level was increased by 1.5-fold in patients with colorectal carcinoma compared to those in healthy controls. When HMGB1 and CEA levels were compared, HMGB1 had similar efficacy as CEA regarding cancer detection (the sensitivity was 20.1% for HMGB1 vs. 25.6% for CEA, and the specificity was 96% for HMGB1 vs. 90.7% for CEA). Moreover, the diagnostic accuracy of HMGB1 for stage I cancer was significantly higher than that of CEA (sensitivity: 41.2% vs. 5.9%; specificity: 96% vs. 90.7). When we combined HMGB1 and CEA, the overall diagnostic sensitivity was higher than that of CEA alone (42% vs. 25.6%), and the diagnostic sensitivity for stage I was also elevated (47% vs. 5.9%). However, the prognosis of patients was not related with serum HMGB1 concentrations. Our findings indicate that serum HMGB1 levels are increased in a subset of colorectal carcinomas, suggesting their potential utility as a supportive diagnostic marker for colorectal carcinomas.

## Introduction

High mobility group (HMG) proteins primarily reside in the nucleus and regulate gene expression by binding to DNA without any sequence specificity [1]. One such HMG protein, high mobility group box-1 (HMGB1), consists of an 80-amino acid A Box, a B Box, and an acidic carboxyl tail. Similarly as other members of its family, HMGB1 binds to the minor groove of DNA in a non-sequence-specific manner [2], [3] and is involved in DNA structural and transcriptional regulation. Nuclear localization of HMGB1 and its affinity for DNA are reported to be regulated by phosphorylation [4] and acetylation [3], [5].

A relationship has been suggested between HMGB1 and cancer based on findings that HMGB1 regulates the transcription of many cancer genes, such as *E-selectin*, *TNF-α*, *insulin receptor*, and *BRCA1* [6], [7], [8]. Another report suggested that extracellular HMGB1, secreted by necrotic cancer cells, might contribute to cancer cell survival, proliferation, and invasion [9]. The association of HMGB1 overexpression and poor prognosis has been reported in cancer patients [9], [10], [11], [12], [13]. In addition to these reports, recent evidence demonstrated that mice with chemically induced colitis exhibited elevated levels of serum HMGB1, and antibody-mediated neutralization of serum HMGB1 decreased the frequency of cancer formation [11]. All of these results suggest that HMGB1 is an important mediator for cancer transformation, cancer growth, and invasion.

HMGB1 secretion and its binding to cell membrane receptors including the receptor for advanced glycation end products (RAGE) appear to be important in cancer progression [14]. The secretion of HMGB1 has been reported in the cells of several malignancies including glioma, colon cancer, lung cancer, and melanoma [15], [16], [17], [18]. In addition to these reports, HMGB1 has been detected in the sera of patients with various cancers, including cervical, lung, gastric, and liver cancers [19], [20], [21], [22], [23]. Despite the detection of HMGB1 in the sera of cancer patient, the clinical evaluation of HMGB1 was not performed in these cancers except for gastric and cervical cancer. The diagnostic sensitivity and specificity of HMGB1 were; 71% and 67% in gastric cancer and 71.6% and 78% in cervical cancer, respectively. Although HMGB1 overexpression has been reported in colon cancer by the percentage over 90% [17], [24], the serum level of HMGB1 and its clinical significance have not been reported. To evaluate the diagnostic significance of secreted HMGB1 *in vivo*, we performed an ELISA assay using blood samples from 219 colorectal carcinoma patients and compared the values to those of 75 non-cancerous controls. We herein demonstrated that HMGB1 is present in the blood of colorectal carcinoma patients, suggesting its utility in the early diagnosis of colorectal carcinomas.

## Methods

### Cells and media

Eleven cell lines, including a cell line derived from normal colonic mucosa (CCD18Co) and 10 cell lines derived from colon cancers (HCT116, LS174T, RKO, DLD-1, LoVo, HCT-8, SW480, SW620, HT-29, and WIDR), were obtained from either the American Type Culture Collection (ATCC, Manassas, VA; http://www.atcc.org) or the Korean Cell Line Bank (KCLB, Seoul, Korea; http://cellbank.snu.ac.kr). Cells were grown in RPMI1640, Dulbecco's modified Eagle's medium, or modified essential medium supplemented with 10% fetal bovine serum (Life Technologies, Grand Island, New York), penicillin, and streptomycin at 37°C in a humidified 5% CO_2_ environment.

### Concentration of secreted proteins

To collect secreted proteins, cells were incubated with serum free media for 1 day before harvesting, and the culture medium was collected and concentrated using iCON 9K concentrators (Thermo Fisher Scientific, Rockford, IL).

### Patient selection and tissue and blood specimen collection

To test serum HMGB1 levels, fresh blood samples from 219 colorectal carcinoma patients and 75 hospital-based healthy controls without evidence of colorectal carcinomas were used. All the samples were collected from 2004 to 2006 and stored in the Liver Cancer Specimen Bank of the Korea Science and Engineering Foundation of the Ministry of Science and Technology at Yonsei University, College of Medicine, Seoul, Korea. Written informed consent was obtained from each patient, and the Institutional Review Board of Severance Hospital and Yonsei University, College of Medicine approved our study (IRB approval No.GR-2007-001). Before blood sample preparation, all of the cancer patients and healthy control subjects underwent colonoscopy and other studies for the detection of malignancy. In the patients who were pathologically confirmed with colorectal carcinoma after colonoscopy, blood samples were collected one day before surgery. The period of blood sample collection was not exceeded two weeks after colonoscopy. In the controls who were confirmed to have no evidence of colon cancer or other tumors, blood samples were collected in the outpatient clinic. Again, the period of blood sample collection was not exceeded two weeks after colonoscopy. The control subjects were considered to have no colorectal precancerous disease such as ulcerative colitis or polyps by colonoscopy and laboratory test. No control subjects have developed cancer during the follow up period. The distribution of age and gender status was almost matched between healthy control subjects and cancer patients, and the demographics were depicted in Table 1. After case selection, all the serum specimens were analyzed without knowledge of disease status.

**Table 1:** The demographics of 75 healthy control subjects and 219 colorectal cancer patients according to age and gender.

	Healthy (%)	CRC (%)
***Age***		
<60	35 (46.7)	101 (46.1)
≥60	40 (53.3)	118 (53.9)
Sum	75 (100)	219 (100)
***Gender***		
Male	47 (63)	137 (62.6)
Female	28 (37)	82 (37.4)
Sum	75 (100)	219 (100)

. All blood samples were delivered to the pathology laboratory within 30 min after collection, and the serum was immediately separated. For blood preparation, 3 cc of blood were collected in a serum separation tube, and the serum was prepared as previously described [25].

### *Western blot analysis*

To compare serum HMGB1 levels between colorectal cancer patients and healthy control subjects, we selected six colorectal carcinoma serum samples and four healthy control samples. Because of the limitation of blood volume in our cases, we selected six colorectal carcinoma serum samples and four healthy control samples according to the volume of collection. The top six most abundant proteins (serum albumin, immunoglobulin G, immunoglobulin A, transferrin, haptoglobin, and antitrypsin) were depleted using MARS (Agilent Technology, Santa Clara, CA) column. Serum (30 µL) was diluted 1∶5 with a proprietary “Buffer A” and loaded onto the MARS column. The unbound fraction was concentrated by ultrafiltration using a Microcon filter (3000-Da cutoff; Millipore, MA). Protein levels in depleted serum samples were measured by the Bradford protein assay method, and 5–10 µg of proteins were separated by 4–12% gradient sodium dodecyl sulfate polyacrylamide gel electrophoresis (SDS-PAGE), transferred to nitrocellulose membranes, and blocked by incubating the membranes at room temperature in tris buffered saline-Tween-20 containing 5% skim milk. Rabbit polyclonal anti-HMGB1 (Abcam, Cambridge, UK) antibody diluted 1∶1000 in blocking buffer was applied to membranes, which were incubated overnight at 4°C. Membranes were washed, incubated for 1 h with horseradish peroxidase-conjugated secondary antibody (Santa Cruz Biotechnology, Santa Cruz, CA), washed again, and developed with ECL-Plus (Amersham Pharmacia Biotech, Uppsala, Sweden).

### Immunohistochemistry

Serial sections from formalin-fixed, paraffin-embedded blocks of 120 colorectal cancer tissues obtained from patients who had not received preoperative chemoradiotherapy were applied to 3-aminopropyltriethoxysilane-coated slides (Sigma, St. Louis, MO). Deparaffinization and rehydration were performed using xylene and alcohol. The slides were pretreated in a microwave oven for antigen retrieval. Sections were incubated for 30 min at room temperature with antibodies against rabbit monoclonal anti-HMGB1 (Epitomics, Burlingame, CA) at a dilution of 1∶250. To block endogenous peroxidase activity, slides were incubated with blocking reagent (DAKO, Glostrup, Denmark) for 5 min before incubation with the primary antibody for 30 min at 25°C. The enzyme-conjugated polymer (EnVision, DAKO) and diaminobenzidine (DAKO) were used as a visualization system and chromogen, respectively. HMGB1 expression was categorized as expressed and negative; cases with definitive epithelial staining in more than 10% of the tumor cells were categorized as expressed, and cases with definite epithelial staining in less than 10% of the tumor cells were categorized as negative.

### ELISA

Serum concentrations of HMGB1 were evaluated by ELISA using the HMGB1 ELISA Kit II (Shino-test, Tokyo, Japan). ELISA was performed according to the manufacturer's instruction. Serum concentrations of CEA were evaluated by chemiluminiscent immunoassay using ADVIA Centaur (Bayer Healthcare LLC, NY, USA). All of the concentrations of HMGB1 and CEA were measured in exactly same samples at a time.

### Statistical analysis

Statistical analysis was performed using MedCalc for Windows, v9.3.3.0 (MedCalc Software, Mariakerke, Belgium). To compare the concentrations of HMGB1 and CEA in patients and healthy controls, we calculated P-values for the average ELISA results by an independent-samples t-test. We constructed receiver operating characteristic (ROC) curves for serum HMGB1 and CEA, either alone or in combination, to assess their diagnostic accuracy in distinguishing colorectal carcinoma patients from normal control subjects. Using the ROC method, we calculated the sensitivity, specificity, error rate, and area under the curve (AUC) to determine the diagnostic accuracy of our findings. The combination of CEA and HMGB1 was evaluated by logistic regression. We also calculated P values to determine whether serum levels of HMGB1 and CEA are related to the histopathologic and clinical features of cancer patients. P values were calculated using the χ^2^ test. The survival outcome was expressed by applying the Kaplan-Meier method, and the log-rank test was used to compare the prognostic significance of individual variables on survival.

## Results

### HMGB1 is overexpressed in colorectal cancer

To compare the expression levels of HMGB1 in colorectal cancer tissues and adjacent normal mucosa, we firstly performed an immunohistochemical experiment using paraffin-embedded colorectal cancer tissues. All the cases should similar results in the colon cancer. HMGB1 was expressed in the nuclei of both normal and tumor cells (Figure S1). However, cytoplasmic HMGB1 expression was exclusively observed in tumor cells. The comparison of HMGB1 expression between the tumors was not significant because all of the tumor cells exhibited the same patterns of strong nuclear expression and weak cytoplasmic expression.

### HMGB1 is detected in the media of colon cancer cells and the blood of cancer patients

**Figure 1:**
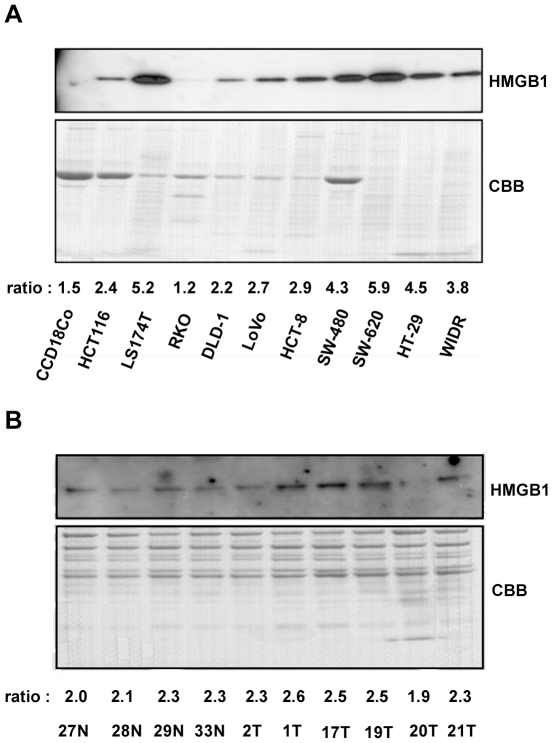
HMGB1 is secreted from colon cancer cells and detected in the blood of cancer patients. HMGB1 in the culture media of colon cancer cells (A) and the sera of colorectal cancer patients (B) exhibited varying degrees of HMGB1 secretion. Culture media were collected, concentrated using a specified column, and the blood samples were depleted of the six most abundant proteins using a MARS column. (A) Normal colon fibroblast cell line CCD18Co did not secrete visible amounts of HMGB1 in the culture media; however most tumor cells secreted HMGB1 with slight differences between tumor cells. Coomassie Brilliant Blue staining indicated that equal amounts of proteins were loaded onto SDS-PAGE gels. (B) Human serum proteins were depleted, and HMGB1 secretion was examined. HMGB1 secretion was observed in both healthy controls and colorectal cancer patients, but elevated serum HMGB1 levels were noted in cancer patients. The numbers provided for the cases match those in our tissue bank database. N represents normal healthy controls, and T represents colorectal carcinoma tumor patients. Coomassie Brilliant Blue staining indicated that equal amounts of proteins were loaded onto SDS-PAGE gels. The ratio of HMGB1∶CBB was calculated by TINA program and depicted under the images.

To identify HMGB1 secretion in colon cancer cells, we measured HMGB1 secretion in several colon cancer cells and compared the levels to those in normal cell line (CCD18Co) by western blot analysis. We observed diverse ranges of HMGB1 secretion levels in the 10 colon cancer cell lines (Figure 1A). In contrast, there was no detectable level of HMGB1 in the media of CCD18Co cells. Based on these results, we have performed the western blot analysis for 10 serum samples to investigate whether serum HMGB1 was detectable in patients and control subjects. We firstly have depleted six abundant proteins in fresh serum by MARS column. HMGB1 was detected in the serum, and HMGB1 levels were increased in colorectal cancer patients compared to those in healthy control subjects. To calculate the ratio between HMGB1 secretion levels in colorectal cancer patients and those in healthy subjects, we quantified the band of western blot images by densitometry, with normalization using CBB (Coomassie Brilliant Blue) staining. By densitometry program, TINA, we have calculated each ratio of HMGB1∶CBB and re-calculated by percentage. The mean value was 150.8±10.3 (mean±SD) in healthy controls and 175.5±23.1 (mean±SD) in cancer patients (P = 0.06). The median value was 151.9 (137.3, 162.0) in healthy controls and 181.6 (140.7, 200.1) in cancer patients (P = 0.1141) by the Wilcoxon rank-sum test. The median values were represented by median (min, max). We demonstrated that serum HMGB1 expression was approximately 1.2-fold higher in colorectal cancer patients than in healthy controls by the mean value, with high diversity between patients (Figure 1B). These results suggest that HMGB1 is a potential diagnostic biomarker for colorectal cancer. For the median values, the difference of these two groups were not significant (P = 0.1141), though both of these levels were followed by normal distribution by Shapiro-Wilk test. In addition to this, the statistical power of these values was 0.4202, which results from small scale samples. To acquire more than 80% of statistical power, more than 12 cases of healthy controls and tumor cases were needed. Although we could not obtain statistically meaningful data, difference between two groups by mean value (1.2 fold increase, p = 0.06 by Welch's t-test) was evident. Based on these data, we performed the HMGB1 value in a large series of clinical samples by ELISA assay.

### Characteristics of the subjects

To test the serum values of HMGB1 and CEA from our collected blood sample sets, we have analyzed the 219 tumor cases according to several clinicopathologic features including age, gender, TNM stage, tumor location, size of tumor mass, status of microsatellite instability, status of recurrence, time of survival, tumor differentiation, and tumor metastasis (Table 2)

**Table 2:** Clinicopathologic features of 219 colorectal cancer patients according to serum HMGB1 levels.

	Serum HMGB1 level (ng/ml)			Serum CEA level (ng/ml)		
Feature	>58.2^a^	≤58.2	*P*	>5^b^	≤5	*P*
*1. Age (y)*			0.4551			0.0066
<60	23	78		17	84	
≥60	21	97		40	78	
*2. Gender*			0.993			0.96
M	28	109		35	102	
F	16	66		22	60	
*3. Stage*			0.0018			<0.0001
I	14	20		3	31	
II	18	65		18	65	
III	7	63		15	55	
IV	5	27		21	11	
*4. Location*			0.5443			0.7789
Colon	25	88		28	85	
Rectum	19	87		29	77	
*5. Size (cm)*			0.1132			0.016
<5	12	73		14	71	
≥5	32	102		43	91	
*6. MSI* c *status*			0.8295			0.2728
High	8	27		6	29	
MSS^d^ and low	36	148		51	133	
*7. Recur status*			0.551			0.127
No Recur	33	131		37	127	
Local	3	6		3	6	
Systemic	8	38		17	29	
*8. Time of survival*			0.4526			<0.0001
<3 years	6	35		22	19	
≥3 years	38	140		35	143	
*9. Differentiation*			0.1349			0.1493
poor	2	11		3	10	
moderate	32	141		50	123	
well	10	23		4	29	
*10. metastasis* e			0.1974			<0.0001
metastatic	10	60		32	38	
nonmetastatic	34	115		25	124	

a The cutoff value of HMGB1 was determined by MedCalc software using a previously published algorithm [44].

b The cutoff value of CEA was determined by the level practically used to diagnose CRC [39], [45], [46].

c MSI, microsatellite-instable cancer.

d MSS, microsatellite-stable cancer.

e The information of tumor metastasis was followed up over 5 years after surgery.

### Increased serum HMGB1 levels in colorectal cancer patients

**Figure 2:**
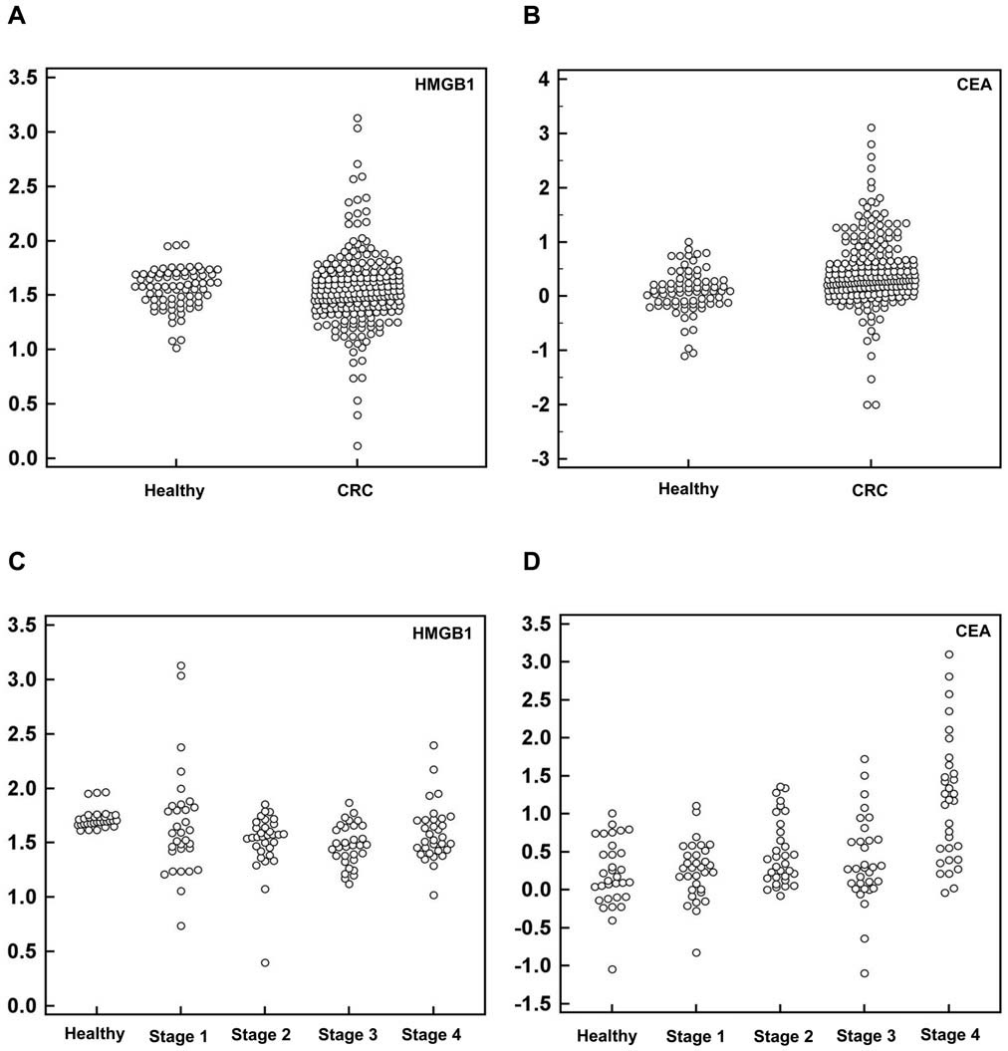
Serum HMGB1 levels are increased in colorectal cancer patients. The sera of 219 cancer patients were screened using HMGB1 ELISA, and the findings were compared with those of 75 non-cancerous healthy controls. Serum CEA levels were also measured in both groups. Each serum value was transferred to natural logarithm to draw a data comparison plot. (A) Serum HMGB1 levels were 1.5-fold higher in cancer patients than in healthy controls (the mean serum concentrations were 58.8±126.2 ng/mL in colorectal cancer patients and 39.7±16.2 ng/mL in control subjects). The P-value was calculated by the Welch's t-test ( = 0.03) (B) Serum CEA levels were elevated in cancer patients compared to those in healthy controls (the mean serum concentrations were 18.3±100.8 ng/mL in patients with colorectal carcinoma and 1.9±1.8 ng/mL in control subjects). The P-value was calculated by the Welch's t-test ( = 0.02) (C) HMGB1 concentrations were depicted according to different tumor stages. (D) CEA concentrations were depicted according to different tumor stages. CEA levels were elevated in advanced tumor stages.

ELISAs were performed to evaluate serum HMGB1 levels in a large series of colorectal cancer patients. We selected serum from colorectal cancer 219 patients and 75 healthy subjects without evidence of carcinoma. The average serum HMGB1 levels were 1.5-fold higher (P = 0.03) in colorectal cancer patients; the mean serum concentration was 58.8±126.2 ng/mL in colorectal cancer patients and 39.7±16.2 ng/mL in control subjects (Figure S2A). The median value was 38.3 (10.3, 92.1) ng/mL for healthy control subject, and 33.3 (1.3, 1350.0) ng/mL for cancer patients by Mann-Whitney test (P = 0.2638). To make all of the values more understandable by reducing the variation, we have replaced the raw data by logarithmic function and the transformed data was shown in Figure 2A. There were also significant differences in the serum levels of CEA between patients with colorectal cancer and control subjects (P = 0.02); the mean serum concentration was 18.3±100.8 ng/mL in patients with colorectal carcinoma and 1.9±1.8 ng/mL in control subjects (Figure S2B). The median value was 1.2 (0.1, 10.2) ng/mL for healthy control subjects, and 2.2 (0, 1274.6) ng/mL in cancer patients (P<0.0001). The values were applied by logarithmic function, and the transformed values were shown in Figure 2B. When we compared the median value of HMGB1 between control subjects and cancer patients, we couldn't identify statistical significance between two groups, implying high variation of the values for HMGB1 in our population may affect the mean value.

To analyze the diagnostic value of serum HMGB1, we analyzed serum HMGB1 levels according to the clinicopathologic features of 219 patients. Serum HMGB1 levels were elevated in stage I cancer compared to those of CEA, whereas serum CEA levels were higher in advanced-stage cancer (Figures S2C and D). The values were applied by logarithmic function, and the transformed values were shown in Figures 2C and D. As expected, the overall diagnostic value according to tumor stage was correlated to these results; the P values of HMGB1 and CEA according to TNM stage were 0.0018 and less than 0.0001, respectively. This result suggests that these two molecules might be complementary for diagnosing colorectal cancer. Unlike to our expectation, our data did not show the close relationship between tumor metastasis and HMGB1 levels, although previous reports suggested the relationship between HMGB1 and tumor metastasis [20]. The elevated CEA levels were closely associated with tumor metastasis (P<0.0001) in accordance with previous reports [26].

**Figure 3:**
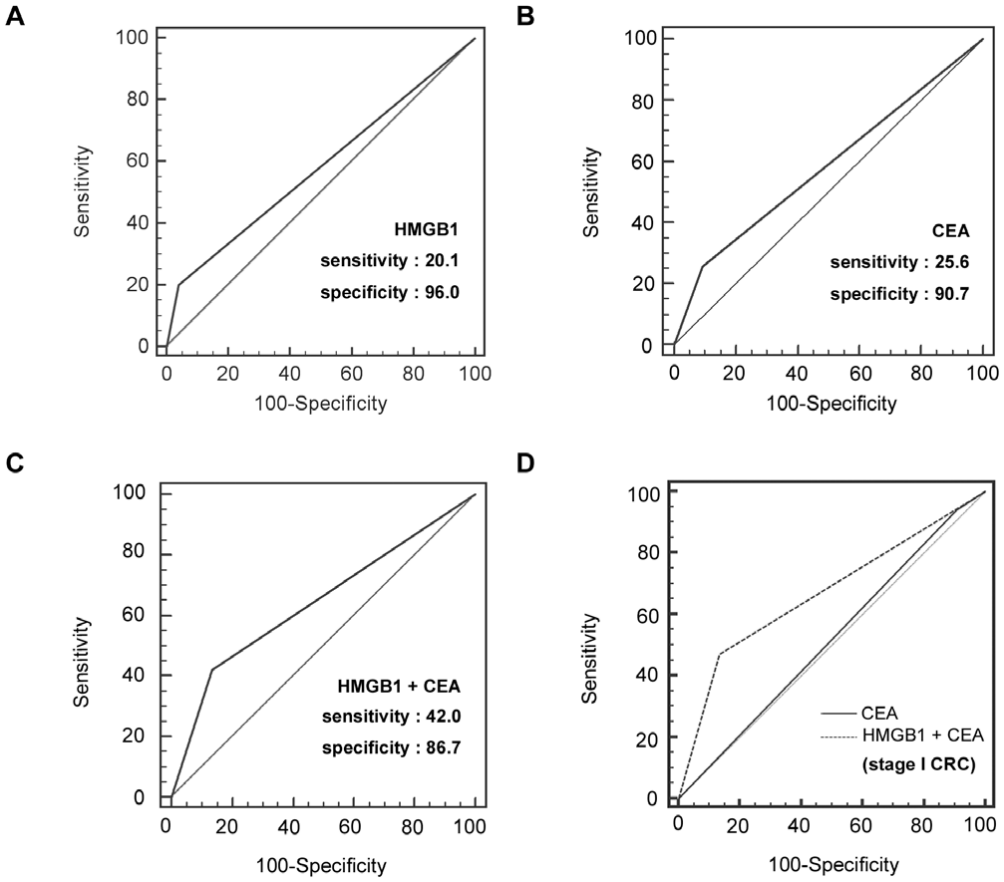
ROC curves generated with serum CEA and HMGB1 levels. To certify the utility of HMGB1 in the diagnosis of colorectal cancer, we used the ROC method to determine cutoff values. (A) ROC curve for HMGB1. At the cutoff value of 58.2 ng/mL, the sensitivity and specificity were 20.1% and 96%, respectively. Overall AUC was 0.580. (B) ROC curve for the CEA. The sensitivity and specificity were 25.6% and 90.7%, respectively. Overall AUC was 0.581. (C) ROC curve for the combination of HMGB1 and CEA. The sensitivity and specificity were 42.0% and 86.7%, respectively. Overall AUC was 0.643. (D) Comparison of combination of HMGB1 and CEA with CEA alone for stage I colorectal cancer. The overall AUC was higher for the combination of these two markers than for CEA alone. All of the reference lines were determined when the AUC was 0.5.

To evaluate the diagnostic value of HMGB1 secretion levels, we used ROC methods to calculate the sensitivity and specificity of HMGB1 and CEA. We determined the 58.2 ng/mL as the cutoff value of HMGB1 using a statistical program (MedCalc). For CEA, we used 5 ng/mL as the cutoff value. We encode serum HMGB1 and CEA levels according to their acquired cutoff value (58.2 ng/mL vs. 5 ng/mL, respectively), and represent ROC curve by re-encoded value. The diagnostic specificity of HMGB1 secretion levels was better than that of CEA, but the sensitivity of HMGB1 was lower (Figure 3A); the sensitivity and specificity of HMGB1 were 20.1% and 96%, respectively, compared to 25.6% and 90.7%, respectively, for CEA (Figure 3B). Because serum HMGB1 levels were high in early stages of colorectal cancer, we combined the diagnostic values of these two markers to increase the accuracy of colorectal cancer diagnosis in early stages. Figure 3C shows that the overall diagnostic value of combination of HMGB1 and CEA had improved sensitivity, but the specificity was not higher than that of CEA or HMGB1 alone (the sensitivity and specificity were 42% and 86.7%, respectively). However, the overall AUC was increased when we combine these two marker; the overall AUCs were 0.580 for HMGB1, 0.581 for CEA, and 0.643 for combination of HMGB1 and CEA, respectively. All of the diagnostic values were summarized in Table 3. These data indicate that the combination of HMGB1 and CEA significantly elevated the diagnostic accuracy for CRC. In our data, the diagnostic sensitivity was significantly increased when these two markers were combined while the specificity was slightly decreased. To assess the diagnostic accuracy by increasing sensitivity, we have compared the sensitivity for HMGB1, CEA, and the combination of these two markers at a fixed specificity. When the specificity was fixed at 90%, the sensitivity of HMGB1, CEA, and the combination of HMGB1 and CEA was slightly elevated. In addition to this, the sensitivity of combination of HMGB1 and CEA was significantly increased compared to HMGB1 or CEA alone at the 80% of specificity (Figure S3). These results indicate that the combination of HMGB1 and CEA has a benefit for the improvement of diagnostic accuracy by increasing diagnostic sensitivity despite the specificity was slightly decreased. In stage I cancers, the diagnostic value of HMGB1 was much higher than that of CEA; the sensitivity and specificity of HMGB1 were 41.2% and 96% and the sensitivity and specificity of CEA were 5.9% and 90.7%, respectively, while the AUC of HMGB1 and CEA in stage I were 0.569 and 0.517, respectively. To compensate the low diagnostic value of CEA in early stage cancer, we have combined those two markers, and the sensitivity of these two markers in combination was much better than that of CEA alone; the sensitivity and specificity of the combination were 47% and 86.7%, respectively (the sensitivity and specificity of CEA were 5.9% and 90.7% for stage I, respectively; Figures 3D). The low sensitivity of CEA in stage I was in accordance with previous report [27]. In our study, only 2 of 34 patients were shown in CEA levels over 5 ng/mL in stage I cancer. These data suggest that the combination of HMGB1 and CEA could increase the diagnostic accuracy for colorectal cancer, especially in early tumor stages.

**Table 3:** Summary of receiver operating curve methods for HMGB1 and CEA.

	Specificity	Sensitivity	Significant level P	Area under the curve
HMGB1	96	20.1	<0.0001	0.580
CEA	90.7	25.6	0.0003	0.581
HMGB1+CEA	86.7	42.0	<0.0001	0.643

### Correlation of HMGB1 and CEA according to the serum levels

**Figure 4:**
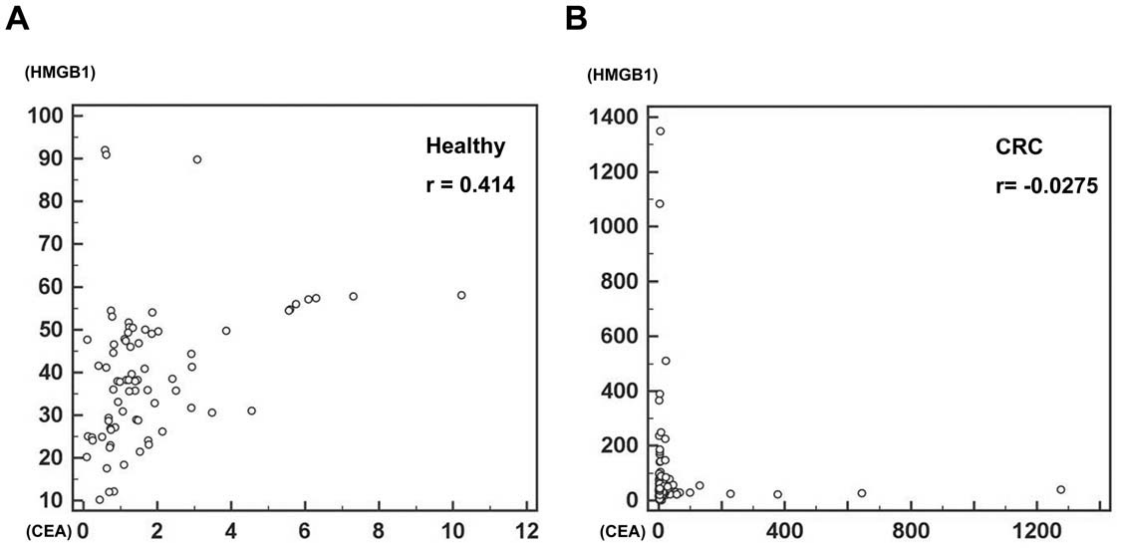
Correlation plot generated with serum CEA and HMGB1 levels. To assess the relationship between serum CEA and HMGB1 levels for the correlation, we used the Spearman's rho method to determine the correlation coefficient r. (A) Correlation plot of 75 healthy subjects for CEA and HMGB1 serum expression levels. The correlation coefficient r was 0.414 (P = 0.0002) for the two values in healthy control group showing these two markers were positively correlated with each other. The X axis represents CEA levels and the Y axis represents HMGB1 levels, respectively. (B) Correlation plot of 219 colorectal carcinoma patients for CEA and HMGB1 serum expression levels. The correlation coefficient r was −0.0275 (P = 0.6858) for the two values in cancer patients demonstrating that these two markers seems to be negatively correlated with each other despite its low accuracy for this tendency. The X axis represents CEA levels and the Y axis represents HMGB1 levels, respectively.

To assess the relationship between serum CEA and HMGB1 levels, we calculated Spearman's correlation coefficient. For 75 healthy controls group, the correlation coefficient constant r was 0.414 (P = 0.0002) demonstrating these two markers were positively correlated with each other in healthy control subjects (Figure 4A). For 219 cancer patients group, the correlation coefficient constant r was -0.0275 (P = 0.6858) demonstrating these two markers seems to be negatively correlated with each other despite its low accuracy by P-value. All of these data suggests that CEA and HMGB1 are correlated with positive tendency in control subject, but does with negative tendency in cancer patients. These results imply that the combination of these two markers can be a good model for the diagnosis colorectal carcinomas.

### Relationship between serum HMGB1 levels and patient survival

We also performed survival analysis according to the serum levels of HMGB1 and CEA by the Kaplan-Meier method. We analyzed the prognostic significance using cutoff values of 58.2 ng/mL for HMGB1 and 5 ng/mL for CEA (Figure S4). In the log rank test, the serum HMGB1 level did not correlate with prognosis (P = 0.336), but the serum CEA level was correlated with the survival rate of colorectal cancer patients (P<0.0001).

## Discussion

In this study, we evaluated serum HMGB1 concentrations in 219 patients with colorectal carcinoma and compared them to the concentrations in healthy control subjects. We identified that the concentration of serum HMGB1 was higher in patients with colorectal cancer than in normal healthy subjects. Our findings suggest that serum HMGB1 levels can be used as a novel diagnostic marker.

Colorectal cancer (CRC) is the third most common malignancy in the world. In Korea, CRC is the fourth leading cause of cancer-related death, and its incidence continues to increase [28]. The risk of recurrence and subsequent death due to CRC is closely related to the stage of the disease at the time of primary diagnosis. Serological biomarkers can be analyzed relatively easily and economically and therefore have the potential to greatly enhance screening acceptance. Various serum markers for CRC are available, among which CEA is the most commonly used marker [29]. However, this marker lacks the sensitivity and specificity needed to screen an average risk population [30]. Therefore, new biomarkers of cancer are needed that will further enhance detection of the disease and trigger a follow-up colonoscopy.

An increasing body of evidence suggests that HMGB1 is associated with tumor metastasis and poor prognosis [9], [10], making HMGB1 an attractive target as a tumor biomarker. Considering the known role of HMGB1, secreted HMGB1 can be involved in tumor metastasis through binding cell surface receptors including RAGE. We previously demonstrated that HMGB1 is translocated to the cytoplasm and secreted by cancer cells [17]. HMGB1 secreted from tumor cells can be involved in tumor progression, particularly metastasis. The evaluation of serum HMGB1 levels is essential for evaluating both the diagnostic significance of HMGB1 in colorectal cancer and the inhibition of cancer progression by blocking serum HMGB1. Several reports indicated that the serum HMGB1 level was elevated in patients with various types of cancer and its diagnostic values were evaluated in some tumors [20], [23]. To our knowledge, this is the first report demonstrating higher serum HMGB1 levels in colorectal cancer patients. The secreted serum HMGB1 is expected to be derived from cancer cells or immune cells in the peritumoral area. In cancer, the roles of HMGB1 have been suggested on the basis of its roles in immune cells. HMGB1 has been reported to be related to poor prognosis in cancer patients [9], [10]. Previous results suggested that HMGB1 is an important mediator for precancerous disease, and therefore, HMGB1 can contribute to the early development of cancer, tumor growth, and invasion of cancer cells [31]. Although HMGB1 plays important roles in immune cells and cancer cells, differences exist in the biologic roles of HMGB1 between cancer and immune cells. First, HMGB1 is acutely translocated and secreted by immune cells in response to TNF-α, IL-1β, or LPS stimulation [32], [33], [34], [35], whereas cancer cells have no known stimuli for translocation and secretion. Instead, cancer cells are known to possess cytoplasmic HMGB1 in its resting state. HMGB1 has known to have diverse functions in cancer including anti-apoptosis, cell-cycle progression, cell growth, invasion, migration, and metastasis [36], [37]. In addition to this, HMGB1 can be strongly secreted by immune activation. Because there are no known tools differentiating secreted HMGB1 between immune cells and cancer cells, we couldn't discriminate the source of HMGB1 secretion in our clinical samples. The secreted HMGB1 from immune cells might cause elevation of HMGB1 from the early stage cancers. Alternatively, HMGB1 was known to be important in malignant cell transformation. In melanoma, HMGB1 is overexpressed in tumor compared to normal melanocyte, leading to malignant transformation and melanoma development [38]. Furthermore, HMGB1 functions as a anti-apoptotic oncoprotein by leading to NF-κB and the target gene product c-IAP (inhibitor of apoptosis) [24]. These reports suggest that HMGB1 could be an oncoprotein for contribution to the tumor development and formation, implying that HMGB1 could be highly secreted in early tumor disease. The identification of the cellular origin of serum HMGB1 will be helpful for the diagnostic utility of serum HMGB1 in the future.

Unlike CEA, elevated serum HMGB1 was also frequently observed in early-stage colorectal cancer. These findings suggest that HMGB1 is useful as a supportive diagnostic marker in colorectal cancer. To evaluate the diagnostic significance of HMGB1, we compared HMGB1 levels with CEA levels and found that the combination of these two markers increases the diagnosis rate of early stage colorectal carcinomas. CEA is a glycoprotein involved in cell adhesion that is normally produced during fetal development. However, its production stops before birth, and CEA is not usually present in the blood of healthy adults. However, it has been found that serum from people with colorectal, gastric, pancreatic, lung, and breast carcinoma has higher levels of CEA than healthy people. In addition, CEA also has reported utility in monitoring the prognosis of tumor patients [39]. The previously reported sensitivity and specificity of CEA were approximately 20% to 40% and 70% to 100%, respectively [40]. In accordance with previous reports, the serum CEA level is related to tumor progression, and, therefore, evaluating serum CEA has limited value in detecting early-stage colorectal carcinomas. We presented the ROC curve for HMGB1 alone in Figure 3A. The sensitivity and specificity of HMGB1 were 20.1% and 96.0%, respectively, and the AUC was 0.580. According to the ROC curve for CEA, the diagnostic sensitivity and specificity were 25.6% and 90.7%, respectively, and the AUC was 0.581 (Figure 3B). We have demonstrated that the diagnostic efficacy was improved using the combination of HMGB1 and CEA compared to that of CEA or HMGB1 alone. The AUC for the combination of HMGB1 and CEA was 0.643, and this value was higher than that of CEA alone (0.581) or HMGB1 alone (0.580). We also demonstrated that the diagnostic efficacy was improved, particularly in earlier stages, as the AUC for the combination of HMGB1 and CEA was 0.669 showing great improvement for AUC result (the AUC for HMGB1 was 0.569 and the AUC for CEA was 0.517, respectively). We, therefore suggest that the serum HMGB1 level is valuable in colorectal carcinoma detection, especially in combination with CEA. In addition, there was evidence that HMGB1 secretion is related to the outcome of chemoradiotherapy [41], but our study only had two patients to investigate this issue. Further investigation of the patients including those who received preoperative chemoradiotherapy might be helpful to validate the diagnostic significance of HMGB1.

The variation of HMGB1 secretion among individuals occurred not only in the cell lines but also in the clinical samples. In the tumor tissues, we found that most of the tumor cells contain variable amount of cytoplasmic HMGB1, while no cytoplasmic HMGB1 was detected in normal epithelial and stromal cells. In the cell line, we have chosen the CCD18Co human colonic fibroblast cell line as a normal control cell line because HMGB1 was scarcely translocated and secreted in this cell line in our previous study [17], [42]. In contrast to the non-neoplastic cell lines, differences in the secretion of HMGB1 were present and may be related to the diverse functions of HMGB1 [33]. RKO cell line among the cancer cells showed very low secretion of HMGB1 and this result corresponds to our previous result [42]. The specific mechanism for this low HMGB1 secretion is not known. HMGB1 levels were extremely high in two patients (1.4 and 1.1 µg/mL, respectively). To assess the effect of these two cases, we reanalyzed the data after excluding these two cases. When we excluded these two cases, the average value of HMGB1 was 1.2-fold higher in healthy control subjects. This changed the diagnostic values of the ROC results: the sensitivity and specificity of HMGB1 were 54.4% and 61.3%, respectively. Despite the reduction of specificity, HMGB1 still exhibits significantly high sensitivity than CEA (sensitivity, 25.8%; specificity 90.7%), thus suggesting that HMGB1 is a useful biomarker to improve the diagnostic sensitivity.

Although high and widespread overexpression of HMGB1 is found in tumor cells [17], [22], only 20% of all patients had serum HMGB1 levels higher than the cutoff value of ROC curve (58.2 ng/mL). The reason for such difference could be explained by a diverse source of serum HMGB1. HMGB1 is secreted from cancer cells and inflammatory cells. This may results in false-positive elevation of HMGB1 in control subjects. To validate a more accurate diagnostic value of HMGB1, a large scale study including inflammatory disease should be performed in the future. We have analyzed the colon cancer patient survival according to the serum HMGB1 level. We could not find any correlation between serum HMGB1 level and patient survival, whereas serum CEA level was correlated to poor patient survival. Serum CEA levels increased with increasing TNM stage, implying that CEA was related to higher stages of colorectal cancer. Although HMGB1 was reported to be related with poor prognosis in colorectal carcinoma tissues in an earlier report [43], we could not compare HMGB1 levels in cancer tissue by immunohistochemistry because most of the tumor cells exhibited HMGB1 overexpression. In the survival analysis using serum HMGB1 levels, no correlation was found between serum HMGB1 levels and patient survival. Further study should be performed to verify the relationship between serum HMGB1 levels and prognostic significance.

## Supporting Information

Figure S1 **HMGB1 expression in colorectal cancer tissues.** A representative colorectal cancer tissue was stained with anti-HMGB1 and counterstained with hematoxylin. HMGB1 was expressed in both tumor cells and the surrounding normal cells. Magnified images are shown on the right part of the figure, which indicated that HMGB1 expression was restricted to the nuclei of normal mucosal cells, whereas cytoplasmic HMGB1 expression was evident in tumor cells.
(TIF)

Figure S2 **Serum HMGB1 levels are increased in colorectal cancer patients.** The sera of 219 cancer patients were screened using an HMGB1 ELISA, and the findings were compared with those of 75 non-cancerous healthy controls. Serum CEA levels were also measured in both groups. (A) Serum HMGB1 levels were 1.5-fold higher in cancer patients than in healthy controls (the mean serum concentration was 58.8±126.2 ng/mL in colorectal cancer patients and 39.7±16.2 ng/mL in control subjects). P-value was calculated by Welch's t-test ( = 0.03) (B) Serum CEA levels were elevated in cancer patients compared to those in healthy control (the mean serum concentration was 18.3±100.8 ng/mL in patients with colorectal carcinoma and 1.9±1.8 ng/mL in control subjects). P-value was calculated by Welch's t-test ( = 0.02) (C) HMGB1 concentrations were depicted according to different tumor stages. (D) CEA concentrations were depicted according to different tumor stages. CEA levels were elevated in advanced tumor stages.
(TIF)

Figure S3 **The comparison of ROC curve at a fixed specificity.** To analyze the impact on the increase of diagnostic accuracy by increasing diagnostic sensitivity, we have analyzed the diagnostic sensitivity at a fixed specificity. (A) The ROC curve for the HMGB1. The lines depicted on the ROC curve showed the sensitivity of y axis at a fixed specificity of 80% (**) and 90% (*), respectively. (B) The ROC curve for the CEA. The lines depicted on the ROC curve showed the sensitivity of y axis at a fixed specificity of 80% (**) and 90% (*), respectively. (C) The ROC curve for the combination of HMGB1 and CEA. The lines depicted on the ROC curve showed the sensitivity of y axis at a fixed specificity of 80% (**) and 90% (*), respectively.
(TIF)

Figure S4 **Survival analysis for colorectal cancer patients according to serum HMGB1 levels.** The survival rate of colon cancer patients according to serum HMGB1 or CEA secretion was analyzed by the Kaplan-Meier method. (A) The prognostic significance of CEA at the cutoff level of 5 ng/mL. According to the log rank test, serum CEA levels were predictive of survival for colorectal cancer patients (P<0.0001). (B) The prognostic significance of HMGB1 at the cutoff level of 58.2 ng/mL. According to the log rank test, serum HMGB1 levels were not predictive of prognosis (P = 0.336).
(TIF)

## References

[1] Goodwin GH, Sanders C, Johns EW (1973). A new group of chromatin-associated proteins with a high content of acidic and basic amino acids.. Eur J Biochem.

[2] Bustin M (1999). Regulation of DNA-dependent activities by the functional motifs of the high-mobility-group chromosomal proteins.. Mol Cell Biol.

[3] Weir HM, Kraulis PJ, Hill CS, Raine AR, Laue ED (1993). Structure of the HMG box motif in the B-domain of HMG1.. EMBO J.

[4] Youn JH, Shin JS (2006). Nucleocytoplasmic shuttling of HMGB1 is regulated by phosphorylation that redirects it toward secretion.. J Immunol.

[5] Bonaldi T, Talamo F, Scaffidi P, Ferrera D, Porto A (2003). Monocytic cells hyperacetylate chromatin protein HMGB1 to redirect it towards secretion.. EMBO J.

[6] Thanos D, Maniatis T (1992). The high mobility group protein HMG I(Y) is required for NF-kappa B-dependent virus induction of the human IFN-beta gene.. Cell.

[7] Baldassarre G, Battista S, Belletti B, Thakur S, Pentimalli F (2003). Negative regulation of BRCA1 gene expression by HMGA1 proteins accounts for the reduced BRCA1 protein levels in sporadic breast carcinoma.. Mol Cell Biol.

[8] Fashena SJ, Reeves R, Ruddle NH (1992). A poly(dA-dT) upstream activating sequence binds high-mobility group I protein and contributes to lymphotoxin (tumor necrosis factor-beta) gene regulation.. Mol Cell Biol.

[9] Kuniyasu H, Chihara Y, Kondo H, Ohmori H, Ukai R (2003). Amphoterin induction in prostatic stromal cells by androgen deprivation is associated with metastatic prostate cancer.. Oncol Rep.

[10] Tarbe N, Evtimova V, Burtscher H, Jarsch M, Alves F (2001). Transcriptional profiling of cell lines derived from an orthotopic pancreatic tumor model reveals metastasis-associated genes.. Anticancer Res.

[11] Maeda S, Hikiba Y, Shibata W, Ohmae T, Yanai A (2007). Essential roles of high-mobility group box 1 in the development of murine colitis and colitis-associated cancer.. Biochem Biophys Res Commun.

[12] Leman ES, Madigan MC, Brunagel G, Takaha N, Coffey DS (2003). Nuclear matrix localization of high mobility group protein I(Y) in a transgenic mouse model for prostate cancer.. J Cell Biochem.

[13] Dolde CE, Mukherjee M, Cho C, Resar LM (2002). HMG-I/Y in human breast cancer cell lines.. Breast Cancer Res Treat.

[14] Taguchi A, Blood DC, del Toro G, Canet A, Lee DC (2000). Blockade of RAGE-amphoterin signalling suppresses tumour growth and metastases.. Nature.

[15] Candolfi M, Yagiz K, Foulad D, Alzadeh GE, Tesarfreund M (2009). Release of HMGB1 in response to proapoptotic glioma killing strategies: efficacy and neurotoxicity.. Clin Cancer Res.

[16] Ito N, DeMarco RA, Mailliard RB, Han J, Rabinowich H (2007). Cytolytic cells induce HMGB1 release from melanoma cell lines.. J Leukoc Biol.

[17] Kang HJ, Lee H, Choi HJ, Youn JH, Shin JS (2009). Non-histone nuclear factor HMGB1 is phosphorylated and secreted in colon cancers.. Lab Invest.

[18] Lim SC, Choi JE, Kim CH, Duong HQ, Jeong GA (2007). Ethyl pyruvate induces necrosis-to-apoptosis switch and inhibits high mobility group box protein 1 release in A549 lung adenocarcinoma cells.. Int J Mol Med.

[19] Cheng BQ, Jia CQ, Liu CT, Lu XF, Zhong N (2008). Serum high mobility group box chromosomal protein 1 is associated with clinicopathologic features in patients with hepatocellular carcinoma.. Dig Liver Dis.

[20] Chung HW, Lee SG, Kim H, Hong DJ, Chung JB (2009). Serum high mobility group box-1 (HMGB1) is closely associated with the clinical and pathologic features of gastric cancer.. J Transl Med.

[21] Naumnik W, Nilklinska W, Ossolinska M, Chyczewska E (2009). Serum levels of HMGB1, survivin, and VEGF in patients with advanced non-small cell lung cancer during chemotherapy.. Folia Histochem Cytobiol.

[22] Shang GH, Jia CQ, Tian H, Xiao W, Li Y (2009). Serum high mobility group box protein 1 as a clinical marker for non-small cell lung cancer.. Respir Med.

[23] Sheng X, Du X, Zhang X, Li D, Lu C (2009). Clinical value of serum HMGB1 levels in early detection of recurrent squamous cell carcinoma of uterine cervix: comparison with serum SCCA, CYFRA21-1, and CEA levels.. Croat Med J.

[24] Volp K, Brezniceanu ML, Bosser S, Brabletz T, Kirchner T (2006). Increased expression of high mobility group box 1 (HMGB1) is associated with an elevated level of the antiapoptotic c-IAP2 protein in human colon carcinomas.. Gut.

[25] Dudek AZ, Mahaseth H (2005). Circulating angiogenic cytokines in patients with advanced non-small cell lung cancer: correlation with treatment response and survival.. Cancer Invest.

[26] Leconte A, Garambois V, Ychou M, Robert B, Pourquier D (1999). Involvement of circulating CEA in liver metastases from colorectal cancers re-examined in a new experimental model.. Br J Cancer.

[27] Fakih MG, Padmanabhan A (2006). CEA monitoring in colorectal cancer. What you should know.. Oncology (Williston Park).

[28] Sung JJ, Lau JY, Goh KL, Leung WK (2005). Increasing incidence of colorectal cancer in Asia: implications for screening.. Lancet Oncol.

[29] Kim HJ, Yu MH, Kim H, Byun J, Lee C (2008). Noninvasive molecular biomarkers for the detection of colorectal cancer.. BMB Rep.

[30] Bast RC, Ravdin P, Hayes DF, Bates S, Fritsche H (2001). 2000 update of recommendations for the use of tumor markers in breast and colorectal cancer: clinical practice guidelines of the American Society of Clinical Oncology.. J Clin Oncol.

[31] Tang D, Kang R, Zeh HJ, Lotze MT (2010). High-mobility group box 1 and cancer.. Biochim Biophys Acta.

[32] Bianchi ME, Manfredi AA (2007). High-mobility group box 1 (HMGB1) protein at the crossroads between innate and adaptive immunity.. Immunol Rev.

[33] Lotze MT, Tracey KJ (2005). High-mobility group box 1 protein (HMGB1): nuclear weapon in the immune arsenal.. Nat Rev Immunol.

[34] Rovere-Querini P, Capobianco A, Scaffidi P, Valentinis B, Catalanotti F (2004). HMGB1 is an endogenous immune adjuvant released by necrotic cells.. EMBO Rep.

[35] Semino C, Angelini G, Poggi A, Rubartelli A (2005). NK/iDC interaction results in IL-18 secretion by DCs at the synaptic cleft followed by NK cell activation and release of the DC maturation factor HMGB1.. Blood.

[36] Ellerman JE, Brown CK, de Vera M, Zeh HJ, Billiar T (2007). Masquerader: high mobility group box-1 and cancer.. Clin Cancer Res.

[37] Evans A, Lennard TW, Davies BR (2004). High-mobility group protein 1(Y): metastasis-associated or metastasis-inducing?. J Surg Oncol.

[38] Poser I, Golob M, Buettner R, Bosserhoff AK (2003). Upregulation of HMG1 leads to melanoma inhibitory activity expression in malignant melanoma cells and contributes to their malignancy phenotype.. Mol Cell Biol.

[39] Wanebo HJ, Rao B, Pinsky CM, Hoffman RG, Stearns M (1978). Preoperative carcinoembryonic antigen level as a prognostic indicator in colorectal cancer.. N Engl J Med.

[40] Holten-Andersen MN, Christensen IJ, Nielsen HJ, Stephens RW, Jensen V (2002). Total levels of tissue inhibitor of metalloproteinases 1 in plasma yield high diagnostic sensitivity and specificity in patients with colon cancer.. Clin Cancer Res.

[41] Apetoh L, Ghiringhelli F, Tesniere A, Obeid M, Ortiz C (2007). Toll-like receptor 4-dependent contribution of the immune system to anticancer chemotherapy and radiotherapy.. Nat Med.

[42] Lee H, Shin N, Song M, Kang UB, Yeom J (2010). Analysis of nuclear high mobility group box 1 (HMGB1)-binding proteins in colon cancer cells: clustering with proteins involved in secretion and extranuclear function.. J Proteome Res.

[43] Yao X, Zhao G, Yang H, Hong X, Bie L (2010). Overexpression of high-mobility group box 1 correlates with tumor progression and poor prognosis in human colorectal carcinoma.. J Cancer Res Clin Oncol.

[44] DeLong ER, DeLong DM, Clarke-Pearson DL (1988). Comparing the areas under two or more correlated receiver operating characteristic curves: a nonparametric approach.. Biometrics.

[45] Chen YD, Zheng S, Yu JK, Hu X (2004). Artificial neural networks analysis of surface-enhanced laser desorption/ionization mass spectra of serum protein pattern distinguishes colorectal cancer from healthy population.. Clin Cancer Res.

[46] Moertel CG, Fleming TR, Macdonald JS, Haller DG, Laurie JA (1993). An evaluation of the carcinoembryonic antigen (CEA) test for monitoring patients with resected colon cancer.. JAMA.

