# Supplementary figures and images for: Diagnostic Significance of Serum HMGB1 in Colorectal Carcinomas
Source: PLoS One. 2012 Apr 4;7(4):e34318. doi: 10.1371/journal.pone.0034318 (PMC3319566; doi:10.1371/journal.pone.0034318)

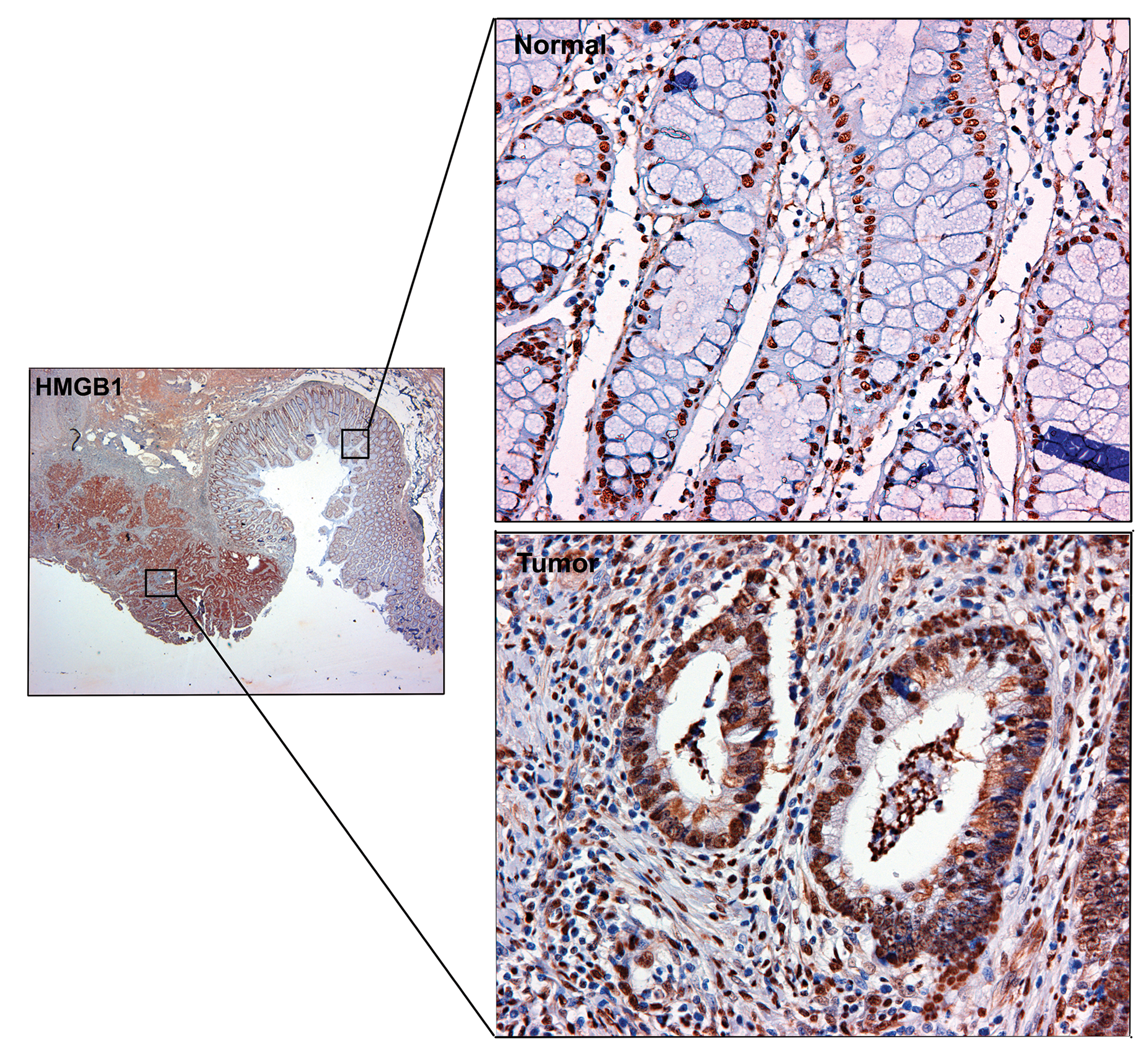

Supplement: Figure S1 — HMGB1 expression in colorectal cancer tissues. A representative colorectal cancer tissue was stained with anti-HMGB1 and counterstained with hematoxylin. HMGB1 was expressed in both tumor cells and the surrounding normal cells. Magnified images are shown on the right part of the figure, which indicated that HMGB1 expression was restricted to the nuclei of normal mucosal cells, whereas cytoplasmic HMGB1 expression was evident in tumor cells. (TIF) [file pone.0034318.s001.tif]
